# Effect of Aluminum Nanostructured Electrode on the Properties of Bulk Heterojunction Based Heterostructures for Electronics

**DOI:** 10.3390/nano12234230

**Published:** 2022-11-28

**Authors:** Oana Rasoga, Carmen Breazu, Marcela Socol, Ana-Maria Solonaru, Loredana Vacareanu, Gabriela Petre, Nicoleta Preda, Florin Stanculescu, Gabriel Socol, Mihaela Girtan, Anca Stanculescu

**Affiliations:** 1 Laboratory of Optical Processes in Nanostructured Materials, National Institute of Materials Physics, 405A Atomistilor Street, P.O. Box MG-7, 077125 Magurele, Romania; 2 Electroactive Polymers and Plasmochemistry, Petru Poni Institute of Macromolecular Chemistry, 41A Gr. Ghica Voda Alley, 700487 Iasi, Romania; 3 Faculty of Physics, University of Bucharest, 405 Atomistilor Street, P.O. Box MG-11, 077125 Magurele, Romania; 4 Optical Processes in Nanostructured Materials Laboratory, National Institute for Laser, Plasma and Radiation Physics, 409 Atomistilor Street, P.O. Box MG-36, 077125 Magurele, Romania; 5 Laboratoire LPHIA, Université d’Angers, LUNAM, 2 Bd. Lavoisier, 49045 Angers, France

**Keywords:** Al nanostructures, nano-patterning, UV-Nanoimprint Lithography, organic heterostructures, MAPLE, bulk heterojunction, non-fullerene acceptors, poly(arylenevinylene)s donors

## Abstract

The properties of organic heterostructures with mixed layers made of arylenevinylene-based polymer donor and non-fullerene perylene diimide acceptor, deposited using Matrix Assisted Pulsed Laser Evaporation on flat Al and nano-patterned Al electrodes, were investigated. The Al layer electrode deposited on the 2D array of cylindrical nanostructures with a periodicity of 1.1 µm, developed in a polymeric layer using UV-Nanoimprint Lithography, is characterized by an inflorescence-like morphology. The effect of the nanostructuring on the optical and electrical properties was studied by comparison with those of the heterostructures based on a mixed layer with fullerene derivative acceptor. The low roughness of the mixed layer deposited on flat Al was associated with high reflectance. The nano-patterning, which was preserved in the mixed layer, determining the light trapping by multiple scattering, correlated with the high roughness and led to lower reflectance. A decrease was also revealed in photoluminescence emission both at UV and Vis excitation of the mixed layer, with the non-fullerene acceptor deposited on nano-patterned Al. An injector contact behavior was highlighted for all Al/mixed layer/ITO heterostructures by I-V characteristics in dark. The current increased, independently of acceptor (fullerene or non-fullerene), in the heterostructures with nano-patterned Al electrodes for shorter conjugation length polymer donors.

## 1. Introduction

Starting as an alternative for improving the performance of solar cells [1,2,3], mixed layers (bulk heterojunctions) are now considered for applications in other organic devices, such as Organic Field Effect Transistors (OFET) [4,5] and organic phototransistors (OPTs) [6,7], organic photodetectors [8,9] and organic photodiodes (OPDs) for visible light communication (VLC) [10]. Recently, tri-phase bulk heterojunctions have been proposed as active layers in organic photodetectors [11,12] and bulk heterojunctions as the active material in resistive random-access memories (RRAMs) [13]. The response of bulk-heterojunction-based organic photodiode to X-ray irradiation has also been investigated [14].

In the classical bi-layer organic heterostructure, the injection and transport of the charge carriers is limited by the short diffusion length of the exciton correlated with the reduced interfacial contact area between donor and acceptor. This drawback can be overcome using an active layer from a mixture between the donor and acceptor component as a bulk heterojunction [15,16]. For example, in the case of solar cells and photodetectors, the photogenerated excitons do not travel long distances to arrive at donor–acceptor interfaces for dissociation. In the thickness of the photoactive layer, the electrons and holes are separated, the active region is extended in the whole volume of the layer and the quantum efficiency can be improved [15]. Thus, a device structure based on a mixed active layer may increase the probability that the exciton reaches a donor–acceptor interface, dissociate and generate free charge carriers within its short (~10 nm) diffusion length. Additionally, the molecular packing and crystallite size of both components within their domain must be optimized. The purpose is to assure an efficient diffusion of the exciton, maximum charge carrier mobility and transfer of the electrons and holes to the electrodes. In the case of electronic devices such as OFETs, the use of both types of charge carriers and the ambipolar transport, with balanced n- and p-type conduction, in the channel material assures good performances [16]. The ambipolar channel material can be obtained by mixing the donor and acceptor components in appropriate ratios and making bulk heterojunction (BHJ) films. Both donor and acceptor in the mixed layer are in contact with the gate dielectric, and at the at the interface donor–acceptor mixed layer/dielectric layer, transport channels are created for both charge carriers [17].

A new type of bulk structure of the active layer has been recently proposed: the interdigitated heterojunction (IHJ), characterized by a distance between pillars shorter than the exciton diffusion length, which determines a large bulk donor–acceptor interface and as a result an efficient exciton dissociation. Thus, it reduces charge recombination and the direct pathway of the generated electrons and holes through the electrodes, which has an effect on the device’s performance [18]. 

In this context, an important step in the optimization of the device’s performance is represented by the selection of adequate donor–acceptor pairs to assure a bicontinuous donor–acceptor composite, which maximizes the interfacial area between the two components. 

The use of polymers in organic heterostructures has opened new perspectives to organic electronics and opto-electronics because of the flexible, lightweight low-cost devices, which can be processed from solutions. The most investigated polymeric donors are based on thiophene and acceptors on fullerene derivatives (e.g., [6,6]-phenyl C_61_ butyric acid methyl ester/PCBM). Lately, significant efforts have been devoted to finding new polymers with adequate properties to be used as donors in bulk heterojunctions.

The proposed p-type conduction donors are poly(arylenevinylene)s, such as poly(N-(2-ethylhexyl)-2,7-carbazolylene-vinylene)/AMC31 and poly(N-(2-ethylhexyl)-3,6-carbazolylene-vinylene)/AMC30 containing carbazole units substituted at 2,7- and 3,6-positions, respectively. These polymers are characterized by good absorption of the radiation from the UV region and blue-green region of the Vis spectrum (wavelength < 450 nm for AMC30 and <540 nm for AMC31) [19]. The band gap estimated from the fundamental absorption edge indicated higher value for the polymer containing carbazole-substituted units in position 3,6 (E_g,AMC30_ = 2.74 eV) compared to the polymer containing carbazole-substituted units in position 2,7 (E_g,AMC31_ = 2.10 eV) [19,20]. Thus, the phenyl group rings linked in para position lead to an extended conjugation along the backbone, without the participation of nitrogen atoms [19]. Additionally, the vinylene segments determine a good delocalization of the π-electrons in solid state.

Because of the limitations shown by fullerene materials, such as weak solubility in common solvents and weak light absorption, increased attention has been paid to find acceptor materials to replace fullerene. In the field of bulk heterojunction organic solar cells, an important research topic is represented by non-fullerene acceptors and recent developments have assured an increased power conversion efficiency exceeding 20% [21,22,23,24]. Non-fullerene acceptors show very good optoelectronic properties, including wide and strong absorption, easy adjustable energy level and stable morphology [23]. Special attention has been paid to investigation of low-bandgap non-fullerene acceptors [25,26], non-fullerene acceptor energy loss by large non-radiative recombination, and on the methods to surpass this limitation [27].

As n-type conduction acceptors, this paper proposes a non-fullerene compound, perylene diimide derivative, featuring strong absorption, low-lying energy level and high electron mobility.

Perylene diimides (PDIs) are promising non-fullerene electron acceptors in mixed layers as they show high absorption and stability. Moreover, the solubility and position of the lowest unoccupied molecular orbital (LUMO) level associated with the electron-deficient character of the compound are tunable material properties [28]. The main drawback of these compounds is related to the planar configuration of these molecules, with a very strong tendency to form aggregates during the film formation. The chemical modification of the proposed perylene diimide derivative, introduced by the use of an aliphatic substituent, assures good solubility and prevents aggregation [29]. Because of the conformational flexibility of alkyl chains, this compound shows an increased solubility [30] without preventing the crystalline packing of the π-conjugated cores [31].

Beside the electronic properties of molecules, the morphological features characterizing the molecular assembling, such as phase separation and crystalline structure, affect the properties of any organic devices based on mixed layers [32]. The morphology of the layer depends on the deposition method and, in the case of solution deposition, it is affected by the solubility of the compounds in the selected solvent.

The PDIs family member N,N′-bis-(1-dodecyl)perylene-3,4,9,10 tetracarboxylic diimide (AMC14) shows a good electron acceptance because of its strong electron-deficient character and forms good-quality layers because of its good solubility in common solvents [33,34].

Some difficulties arising from the use of AMC14 could be related to the position of the LUMO level above the level situated at 3.6 eV, which is attributed to an electron trapping mechanism that dominates in organic semiconductors [35]. In organic semiconductors, this trapping level is attributed to a universal trap center [36] on which the electrons’ recombination occurs. In this case, the transport in the compounds with the LUMO level in a higher position in the energy scale is a trap-limited electron transport [36]. Therefore, for assuring a trap-free electron transport, it is better that the LUMO level is located deeper than 3.6 eV. On the other hand, from theoretical simulations it has been deduced that when the position of the highest occupied molecular orbital (HOMO) level in the organic is lower than 6 eV, the transport will be a trap-limited hole transport [36]. These values of LUMO and HOMO delimit the range where the transport is not limited by charge carrier trapping. Taking into account the higher position in the energy scale of the HOMO energetic levels in the selected donors AMC30 and AMC31 (E_HOMO, AMC30_ = 5.15 eV; E_HOMO, AMC31_ = 5.33 eV [37], a trap-free hole transport is expected in the mixed layer. The position in the energy scale of the acceptor’s LUMO (E_LUMO, AMC14_ = 3.44 eV [38]) suggests a more plausible trap-limited electron transport.

Additionally, a weak hole transfer is expected from AMC14 to AMC31 because of the offset between the HOMO of AMC14 (E_HOMO; AMC14_ = 5.16 eV) [38] and the HOMO of AMC31, and of the energy barrier that must surpass ΔE = E_HOMO,AMC31_−E_HOMO,AMC14_ = 0.17 eV. A better hole transfer is expected from AMC14 to AMC30 because of the close position of the HOMO level in these compounds. On the contrary, the electron transfers from both AMC30 and AMC31 to AMC14 is very plausible because the position of the LUMO level in AMC30 and AMC31 (E_LUMO, AMC30_ = 2.41 eV; E_LUMO, AMC31_ = 3.23 eV) is higher in the energy scale than the position of LUMO in AMC14 (E_LUMO, AMC14_ = 3.44 eV [38]), which favors the electron transport.

The other acceptor proposed for comparison is a functionalized fullerene [6,6]-phenyl C_61_ butyric acid butyl ester, named PCBB or [60]PCB-C4 according to Sigma-Aldrich (Merck KGaA, Darmstadt, Germany), which has already been tested as an acceptor in bulk heterojunction mixed with star-shaped arylenevinylene oligomer as donor [39]. The pendant group attached to fullerene C_60_ modifies the behavior, compared to unsubstituted C_60_, by increasing the solubility. Thus, this fullerene derivative shows the advantages of fullerene, related mostly to the high mobility of the electron determined by the highly conjugated nature of fullerene molecules and, in addition, a better solubility in common solvents because of its long aliphatic tail.

Among the factors with negative impact on the properties of the organic heterostructures are the reflection/scattering of radiation correlated with limited absorption, morphology at the nanometer scale of the active layer, charge carrier recombination, inefficient charge transport, and offset between the energetic levels of donor and acceptor [40]. Thus, to improve the performances of opto-electronic devices, it is necessary to consider two aspects: manipulation of light and increase of charge carrier transport and collection by the electrodes. To reach this aim, the use of nanostructured electrodes represents an alternative. Until now, significant efforts have been spent in using photonic/plasmonic elements in organic photovoltaic devices through different strategies, such as the incorporation of metallic nanoparticles or the patterning of metal electrodes for coupling the incident radiation with the surface plasmon polariton (SPP) modes or waveguide modes that propagate in the plane of the active layer [41]. Previous investigations have highlighted how nano-patterning affects the properties of aluminum (Al) layers [42]. The effect of periodic patterned micro/nanostructures developed on the metal surface, at the interface electrode/organic semiconductor on the properties of small molecule organic bi-layer heterostructures [43], bulk-heterojunction-based organic heterostructures [44], organic photovoltaic devices [45] and nucleobase thin films for optoelectronic applications [46] has also been investigated.

This paper reports some results of the studies on the organic heterostructures Al/donor:acceptor/indium tin oxide (ITO), realized with mixed layers containing a “p” type conduction arylenevinylene-based polymer donor, and an “n” type conduction acceptor, [6,6]-phenyl C_61_ butyric acid butyl ester or perylene tetracarboxidiimide, blended in the weight ratio of 1:2. The optical and electrical properties of these heterostructures were investigated in correlation with the type of acceptor. The optical and electrical properties of the mixed layer obtained by blending perylene tetracarboxidiimide with arylenevinylene monomers were investigated in a previous paper [47].

Moreover, the properties of the heterostructrure with mixed layers deposited on Al flat are analysed by comparison with those of the heterostructures with the same mixed layer, deposited by the same method, on a nanostructured Al electrode.

## 2. Experimental Section

**Figure 1:**
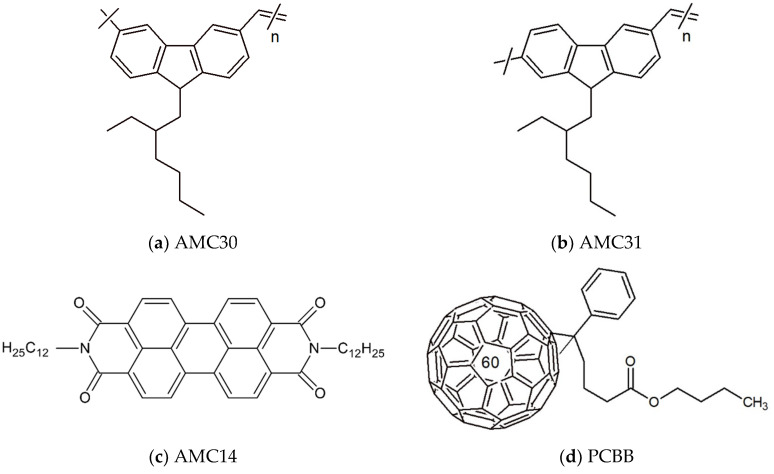
Molecular structure of poly(N-(2-ethylhexyl)-3,6-carbazolylene-vinylene)/AMC30 (**a**); poly(N-(2-ethylhexyl)-2,7-carbazolylene-vinylene)/AMC31 (**b**); N,N′-bis-(1-dodecyl)perylene-3,4,9,10 tetracarboxylic diimide/AMC14 (**c**); [6,6]-phenyl C_61_ butyric acid butyl ester/PCBB (**d**).

The poly(arylenevinylene)s,poly(N-(2-ethylhexyl)-3,6-carbazolylene-vinylene) named AMC30 (Figure 1a) and poly(N-(2-ethylhexyl)-2,7-carbazolylene-vinylene) named AMC31 (Figure 1b) were obtained with Stille Pd(0)-catalyzed coupling polymerization reactions of dibromo-derivatives, (N-(2-ethylhexyl)-2,7-dibromocarbazole for AMC31 and N-(2-ethylhexyl)-3,6-dibromocarbazole for AMC30), with trans-1,2-bis(tributylstannyl) ethane. Both the yellow-colored powder polymer AMC31 [19] and green-colored powder polymer AMC30 [19] have the same structural unit (C_24_H_25_N) and are soluble in 1,2-dichlorobenzene. Details about the preparation of polymers AMC30 and AMC31 were presented in previously published papers [19,20]. For the synthesis of N,N′-bis-(1-dodecyl)perylene-3,4,9,10 tetracarboxylic diimide named AMC14 (Figure 1c), the reaction between 3,4,9,10-tetracarboxylic-perylendianhydride and dodecylamine was used as previously discussed [44]. The acceptor [6,6]-phenyl C_61_ butyric acid butyl ester named PCBB (Figure 1d) was purchased from Sigma-Aldrich (Merck KGaA, Darmstadt, Germany) and used without further purifications.

Beside the identification of new donor (including polymeric ones) and acceptor materials, another way to improve the performances of organic devices could be the improvement of the quality of the mixed layer through the optimization of the active layer preparation method.

We have selected the Matrix Assisted Pulsed Lased Evaporation (MAPLE) method to deposit the mixed layer because it presents advantages related to morphology and thickness control and offer the possibility to prepare films on different substrates, independently of the wetting or non-wetting property [48]. This method does not require ultra-high vacuum and is very flexible to the selection of solvent [49]. By the choice of deposition conditions, it is possible to obtain thin films from a large variety of materials, preserving the chemical and structural particularities (small molecule compounds, polymers, biomaterials, nanomaterials, mixed layers) [50,51,52,53,54,55,56]. This is possible because MAPLE uses some specific deposition conditions for avoiding/minimizing the photo-chemical and photo-thermal damages associated with high energy and temperature at the interaction of laser radiation with the material. Therefore, our materials of interest were dissolved in low weight percentages in a solvent (1,2-dichlorobenzene) characterized by high molecular weight (147 Da) and a good absorption at 248 nm, which is the wavelength of the laser radiation, with the purpose of generating a homogeneous solution. The properties of the solvent and the low concentration favor low fluencies, which are adequate for the deposition of soft materials (organic and bio materials). An important drawback of MAPLE is related to the surface morphology, which is expected to show micron-size droplets in the case of polymer evaporation [57]. However, the low concentration in organic compounds and the conditions in plasma plume are not favorable for the generation of large polymer features such as clusters or aggregates.

1,2-dichlorobenzene was also selected as the solvent because its volatility favors a more ordered morphology of the film deposited from solution compared to the high volatility chloroform generating highly disordered morphologies [58]. This order is favored by the lower degree of polymer chain folding and twisting. Furthermore, AMC14 is characterized by a good solubility in 1,2-dichlorobenzene, which is essential for the morphology of the deposited film.

The deposition equipment was based on a Coherent ComplexPro 205 excimer laser (Coherent LaserSystem GmbH & Co. KG, Gottingen, Germany) [59,60,61,62]. Using a MgF2 lens with a focal length of 300 nm, the laser radiation with a wavelength of 248 nm was focalized on the target. The solution of polymer with AMC14 or PCBB in 1,2 dichlorobenzene was homogenized with magnetic stirring and frozen at the liquid nitrogen temperature for obtaining the solid target, which was evaporated under ultraviolet (UV) irradiation. The target concentration in solute was 3 g/L and the two components of the layers were mixed in the weight ratio of 1:2. To avoid the local heating of the target generated by the laser beam, the target rotated with a frequency of 10 Hz. The local deterioration produced by heating can generate the modification of the local evaporation conditions, thus affecting the quality of the deposited film. The excimer laser spot area was 33 mm^2^ and energy was 79 mJ, corresponding to a fluence of ~210 mJ/cm^2^. The deposition took place with the substrate maintained at room temperature situated 5 cm from the target. The pressure in the deposition chamber was 10^−3^ mbar. All the mixed layers were deposited at 25,000 pulses.

**Figure 2:**
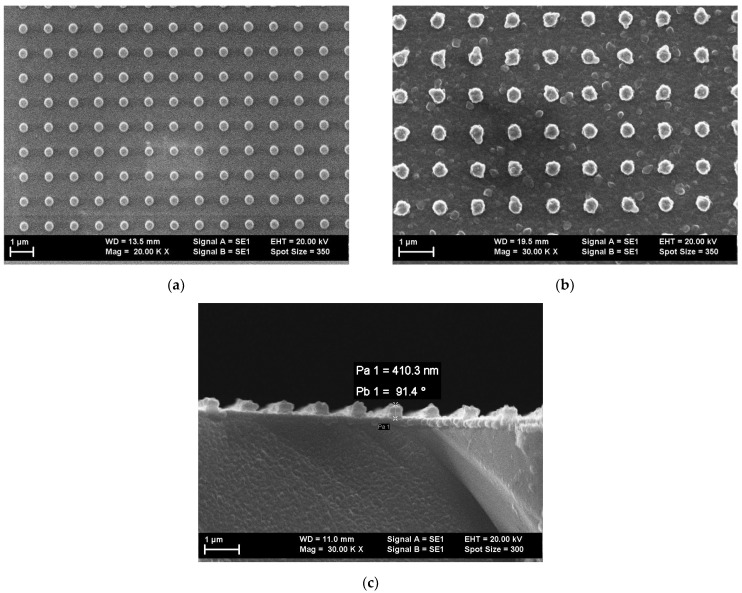
SEM images of: nano-patterned polymeric photoresist (**a**); nano-patterned Al layer (**b**); nano-patterned Al layer cross-section at a sample tilt angle of ~90° (**c**).

The organic heterostructures were realized on single silicon substrates (Si) with an area of 1.8 cm × 1.8 cm, previously cleaned in alcohol isopropyl. Reference samples of the mixed layers were also deposited on glass cleaned in alcohol isopropyl. The flat Al electrode was deposited using sputtering on Si substrate. A two-dimensional (2D) periodic structure with a cylindrical pillar-like shape with a periodicity of ~1.1 µm was realized using UV-Nanoimprint Lithography in the photoresist polymeric layer [42,43,44]. The patterned Al electrode was obtained with the deposition of Al on these structures [42,43]. For this purpose, we used a system containing a Brewer Science Cee 200X Spin Coater (Brewer Science Ltd., Derby, England), an EV Group soft stamp, and an EVG 620 mask aligner. The soft stamp, characterized by a pillar diameter of 700 nm, pillar height of 300 nm and periodicity of 1.1 µm, was the negative of the pattern to be developed in the photoresist layer. The patterning was realized in the following conditions: deposition of photoresist by spin coating at a speed of 2000 rpm and duration of 40 s; thermal treatment of photoresist at 120 °C for 30 s; vacuum contact pressure between the soft stamp and the photoresist of 200 mbar. The photoresist was solidified by exposure to UV radiation for a duration of 90s. After this, the stamp was removed, leaving the desired pattern in the polymeric layer of the photoresist (Figure 2a). More details about the patterning procedure are given in [42,44]. An Al layer with a thickness around 100–120 nm was deposited on top of the nano-patterned photoresist by sputtering using a Bestec UHV-Deposition System (Bestec GmbH, Berlin, Germany) in the following conditions: pressure in the chamber = 1 × 10^−7^ mbar and duration of deposition = 2 h (Figure 2b,c).

The images of the nano-patterned surfaces of both photoresist and Al layers were obtained using Scanning Electron Microscopy (SEM) using a Zeiss EVO 50XVP microscope working at an acceleration voltage = 20 kV and magnification = 20 kX; 30 kX. The Al layer deposited on the nano-patterned photoresist (Figure 2b) was characterized by the presence of clusters of boules (inflorescence-like), this morphology being similar to the morphology already mentioned in a previous paper [42].

The shape of the pillars slightly changed and the edge of the pillars became irregular, because during the deposition the metal atoms attached not only on top but also on the lateral walls and at the bottom of the gap delimited by nanostructures, causing an increase in the area of the pillars’ bases and a decrease in the distance between the walls of two successive nanostructures. The height of the pillars increased from ~300 nm without an Al layer (determined by the stamp) to ~410 nm with an Al layer (Figure 2c), as a positive balance between the thickness of the atom layer deposited on top of the pillars and that of the atom layer deposited at the bottom of the gap which separate the pillars.

The organic heterostructures realized on Si substrates covered by flat aluminum (Al_flat_) and aluminum nano-patterned (Al_nano_) were characterized by optical methods. A Carry 5000 Spectrophotometer (Agilent, Santa Clara, CA, USA) was used to draw the ultra-violet-visible–near-infrared (UV-Vis-NIR) transmission spectra of the mixed layers deposited on glass in the spectral domain 200–1600 nm. With a Perkin-Elmer Lambda 45 UV-Vis, including an integrating sphere with illumination at 8°, the total reflectance spectra was obtained (both specular and diffuse reflectance) in the domain of 350–950 nm of the same mixed layers deposited on Si. An Edinburg Instruments F-900 Spectrofluorometer (Edinburg Instruments Ltd., Livingston, UK) was utilized to draw the photoluminescence emission (PL) spectra at two excitation wavelengths, 335 nm and 435 nm, within the measurement range 400–750 nm with a slit of 2.5. The analysis of Fourier transform infrared (FTIR) spectra was obtained with a Spectrum BX II Perkin Elmer Spectrometer (PerkinElmer LAS (UK)Ltd., Seer Green, Beaconsfield, UK), which offered information about the variation in the chemical composition of the mixed layers and, in consequence, on the preservation or degradation of the chemical structure of the organic compounds.

Information about the surface topography of the mixed layer was obtained using Atomic Force Microscopy (AFM) with a MultiView Nanonics 4000 System (Nanonics Imaging Ltd., Jerusalem, Israel) using tapping in the following conditions: probe diameter = 20 nm, probe frequency = 35 kHz, probe factor of merit 1850; scan area = 20 µm × 20 µm; scan resolution = 256 lines, scan speed = 6.12 lines/s. The WS × M4.0 beta 9.2 (developed by Julio Gómez Herrero & José María Gómez Rodríguez) and Gwyddion 2.47 (developed by D. Necas, P. Klapetek, and coll.) version 2.47 (Free and Open Source software, covered by GNU General Public License), software allowed the evaluation of the statistical parameters of the surface: root-mean-square (RMS), roughness average (RA), skewness (S_SK_) and kurtoisis (S_KU_). The statistical parameters were calculated after the subtraction of the mean plane. Three scans were made on each sample and the values of the surface parameters are the average values of the parameters obtained at each scan. For obtaining relevant images and topographic profiles, the map data were processed by applying a polynomial background removing (n = 2) and smoothed using a Gaussian filter (n = 2).

The indium tin oxide (ITO) top electrode with an area of 1.8 cm × 1.2 cm was deposited on the mixed layer using Pulsed Laser Deposition (PLD), using the same laser and experimental configuration as for MAPLE. We used an ITO bulk solid target (manufacturer Sci Engineer Sci Engineered Materials Inc., Columbus, OH, USA) with the composition: 90 wt. % In_2_O_3_: 10 wt. % SnO_2_. The deposition conditions were: fluence = 1.2 J/cm^2^, number of pulses = 7000, target rotation frequency = 10 Hz, oxygen pressure in the deposition chamber = 1.5 Pa.

The tool used to evaluate the thickness of the layers was an Ambios Technology XP100 profilometer (Ambios Techology Inc., Santa Cruz, CA, USA). The mentioned values were obtained as an average value of three measurements made in different points on each layer. For the ITO layer a thickness around 250 nm was obtained, and for the mixed layer a thickness between 400 nm and 550 nm was obtained. 

**Figure 3:**
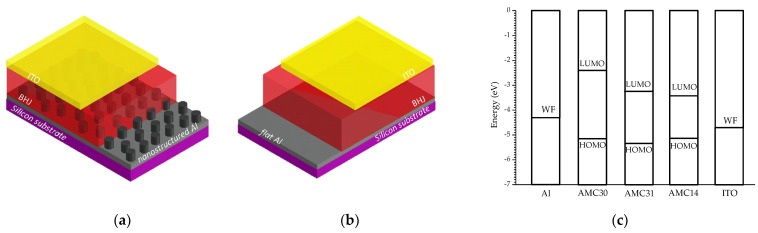
Schematic representation of the organic heterostructure with mixed layer AMC30/AMC31:PCBB/AMC14 on: Al_nano_ electrode (**a**); Al_flat_ electrode (**b**). Energy level diagram with the position of the HOMO, LUMO and work function (WF) level for the components of the heterostructures (**c**).

Current–voltage (I–V) characteristics of the heterostructures built on the Al_flat_ (Figure 3a) and Al_nano_ (Figure 3b) electrodes with a AMC30/AMC31:PCBB/AMC14 mixed layer and an ITO top electrode (the position of the energetic levels of the component layers is mentioned in Figure 3c) were drawn in normal temperature and pressure conditions, in dark, using a system based on a Keithley 2400 Source Meter (Tektronix, Beaverton, OR, USA) assisted by a computer. The measurement configuration was transversal with four contact wires: two on the bottom Al and the other two on the top ITO electrode to eliminate the effect of the contacts.

## 3. Results and Discussions

The optical properties of arylenevinylene polymers are caused by the chemical and electronic structure determined by the conjugation between the carbazole unit and the spacer. This conjugation occurs through a biphenylene unit and shows a longer conjugation path and increased π-electron delocalization, when the carbazole unit is functionalized at the 2,7 position [19,20]. The conjugation takes place between the phenyl units and spacer in the case of 3,6 disubstituted carbazole units. This conjugation is obtained through the lone electron pair localized on the nitrogen atom of the carbazole unit [19,20,36]. 

**Figure 4:**
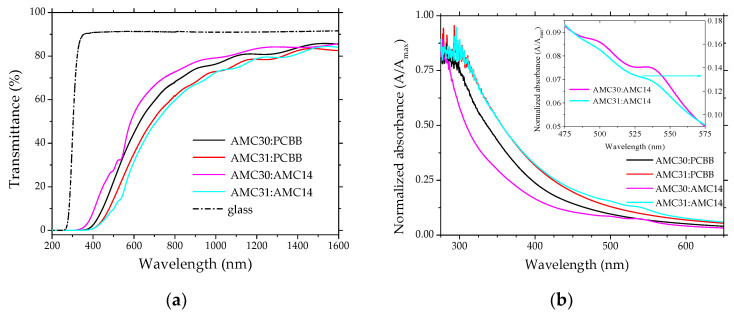
UV-Vis transmission spectra of the AMC30/AMC31:PCBB/AMC14 mixed layers on glass (**a**); normalized absorbance spectra of these mixed layers deposited on glass (**b**). Inset shows the normalized absorbance of the mixed layers in the range 475–575 nm. A = Absorbance; A_max_ = maximum absorbance.

The fundamental absorption edge shows some specific features for the thin films containing AMC14 (Figure 4a,b). This is an absorption mechanism in two steps, with thresholds localized at 495 nm and 535 nm (Figure 4b). The first mentioned absorption peak was better defined in the film containing AMC30 and the second peak in the films containing both AMC30 and AMC31. On the fundamental absorption edge of the samples with PCBB have not been identified additionally thresholds. This means that the two thresholds situated at 495 nm and 535 nm can be associated with AMC14. The fundamental absorption edge was slightly red-shifted for the films with AMC31, independently of acceptor, PCBB or AMC14. This red shift of the absorption edge is in concordance with the red shift shown in solution by AMC31 compared to AMC30, because the absorption wavelength maximum depends on the substitution positions of carbazole units [19]. In conjugated polymers, absorption takes place along the chain backbone [58] and thus the alignment of the polymer chains and the conjugation length affects the UV-Vis measurement. However, in the low-molecular-weight polymer based on the 3,6 disubstituted carbazole, AMC30, the conjugation length 2qs smaller than in the polymer based on the 2,7 disubstituted carbazole, AMC31 [19]. Thus, in our case, the 2,7 positions substitution assures a longer conjugation than the 3,6 positions substitution, explaining the red shift for AMC31. The slightly oscillating shape of both AMC31 and AMC30 absorption spectra in the presence of AMC14 could be associated with π-π* electronic transitions of the perylene chromophores [38].

The red shift in the solid-state spectrum was determined by a more effective packing (π-stacking) determined by π-π overlapping of the polymer chains and more effective intermolecular and intramolecular interactions in polymer AMC31 compared to AMC30, also extending the conjugation length [19,20]. The red shift of the absorption edge was associated with an extended absorption domain for AMC31 (up to approximately 540 nm) compared to AMC30 (up to approximately 450 nm). 

The wide oscillations revealed by the spectra of the mixed layer deposited on glass at wavelengths longer than 800 nm can be correlated with interference phenomenon of the radiation reflected at the interfaces air/mixed layer and mixed layer/glass substrate.

Additionally, the reflection properties of the mixed layer deposited on the metallic electrode covering the Si substrate have also been investigated. The reflectance spectrum is affected by the presence of Al nanostructures, the mixed layer deposited on the Al nano-pattern showing lower reflectance compared to the same mixed layer deposited on Al flat. The radiation is reflected by the bottom flat interface Al/photoresist on the Si substrate and also by the interface air/Al nano-patterned. The incident light was reflected by the surface on top of the pillars and by the surface on the bottom of the gap between the pillars. Supplementary reflections take place from the lateral walls of the gaps delimited by the pillars. These reflected beams interfere and can satisfy the conditions of a constructive or destructive interference. Thus, the interference phenomenon is affected by the presence of nanostructures, causing supplementary reflections and multiple scatterings of radiation. Furthermore, the optical properties of the Al nano-pattern were determined by the resonances of the localized surface plasmons (LSPRs) and light trapping, which is correlated with the enhanced scattering generated by the presence of nanostructures [63]. Thus, the nanostructures favor light trapping, increasing absorption and reducing reflection.

**Figure 5:**
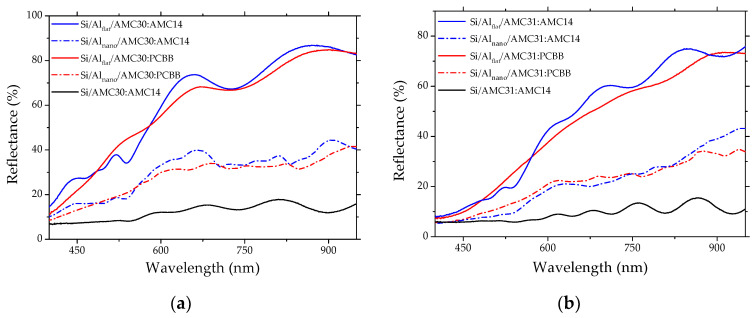
Reflectance spectra of the mixed layers with AMC 30 donor and PCBB or AMC14 acceptor deposited on Al_flat_ and Al_nano_ (**a**). Reflectance spectra of the mixed layers with AMC 31 donor and PCBB or AMC14 acceptor deposited on Al_flat_ and Al_nano_ (**b**). Si/AMC30:AMC14 and Si/AMC31:AMC14 are reference samples.

The reflectance of the mixed layers containing AMC30 and PCBB or AMC14 deposited on Al flat (Figure 5a) was between 60 and 85% for wavelengths over 600 nm, and the reflectance was influenced by the behavior of the metallic film related to the contribution to the dielectric constant of the free electrons. The light trapping by multiple scattering inside nanostructures reduced the reflectance [64,65] of the same mixed layers deposited on Al_nano_, between 30 and 40%, for wavelengths over 600 nm. The reflectance spectrum of the mixed layer AMC30:AMC 14 deposited on Si, without an Al layer, showed lower amplitude and slightly irregular oscillations. The layer containing AMC30 and AMC14 deposited on Al_flat_ showed irregular oscillations, with a slightly higher amplitude compared to the layer containing PCBB, and supplementary oscillations were introduced by the Al nano-patterning. 

The reflectance of the mixed layer containing AMC31 (Figure 5b) was lower than the reflectance of the mixed layer containing AMC30. The reflectance was between 50 and 75 % when the mixed layer was deposited on Al_flat_, and between 25 and 40% when the mixed layer was deposited on Al_nano_, for wavelengths over 600 nm. The mixed layer containing AMC14 deposited on Al_flat_ showed larger oscillations compared to the layer containing PCBB. The nano-patterning of Al introduced a more regular periodicity of the signal obtained from the mixed layer with an AMC31 donor and PCBB acceptor. The reflectance signal of the mixed layer containing an AMC14 acceptor showed oscillations when the layer was deposited on both Si and Si covered by Al_flat_, but in the last case the oscillations were slightly irregular.

Aluminum suffers a natural oxidation process when exposed to air, resulting in an amorphous layer of aluminum oxide (Al_2_O_3_) of 3–5 nm that can influence the optical properties [65]. The presence of the oxide layer affected the position and regularity of the interference’s fringes generated in the thickness of the mixed layer containing AMC14 and AMC30 (Figure 5a) or AMC31 (Figure 5b) deposited on Si covered by Al. Aluminum favors the appearance of plasmonic effects in UV and Vis of the spectrum with relatively low losses, and the oxide layer affects the optical properties, such as intensity and spectral shift of resonances [66].

Our samples deposited on Al_nano_ showed a dip in the reflectance spectrum situated around 820 nm in both mixed films AMC31:PCBB and AMC31:AMC14 (Figure 5b), corresponding to an energy of ~1.5 eV, which is associated with inter-band transitions (absorption) of electrons in Al [65,67]. Other dips in reflectance spectra were situated in the spectral range 500–950 nm: around 540 nm, 680 nm, 760 nm for AMC31:AMC14 and 640, 710, 770 and 915 nm for AMC31:PCBB (Figure 5b). The minima in the reflectance spectrum were correlated with the coupling between the incident wavelength and the surface plasmons excitations. These excitations were localized in the nanostructures developed in Al which are partially filled with the organic molecules of the mixed layer, at the interface Al-organic. A succession of dips was also revealed in the reflectance spectrum of AMC30 mixed with PCBB or AMC14 deposited on Al_nano_ (Figure 5a), but the dip corresponding to inter-band absorption (820 nm) was slightly red-shifted to 830–850 nm. This behavior can be determined by the thickness of the mixed layer, geometrical dimension of the gaps (cavities) between pillars remaining after the deposition of the mixed layer, and thickness of the aluminum oxide layer covering the Al electrode. In general, the reflectance amplitude of the mixed layer deposited on Al_nano_ tended to decrease and the reflectance oscillations were weaker compared to the same mixed layer deposited on Al_flat_. The interference effects between light reflected from inside the cavities and from the top of the film cannot be revealed because of the rough surface of the organic mixed layer. 

The Al_flat_ film showed metallic behavior and therefore the free electrons contributed to the dielectric constant. In the case of Al_nano_, the contribution of the free electrons to the dielectric constant generated Localized Surface Plasmon Resonances (LSPRs), introducing multiple scattering and light-trapping. The conditions related to the dimension/shape of nanostructures (diameter, height) and separation (periodicity) between nanostructures and Al layer thickness are essential for an enhanced light-trapping mechanism [63]. Our 2D array of nanostructures was characterized by a reduced number of structures per unit area, which generated a reduced number of scatterings associated with a low level of light trapping and broad- and shallow-reflectance resonance dips.

Because the mixed layer was prepared with a laser technique using a laser beam with a UV wavelength, it is necessary to verify if the molecular structure was preserved or suffered modification during this process through a photo-degradation mechanism. Therefore, the FTIR spectra of the mixed layer deposited by MAPLE were compared with the FTIR spectra of the mixed layer prepared by drop cast using the same solution as those utilized for the MAPLE target preparation. The drop cast process involves the evaporation in normal conditions of temperature and pressure, and therefore the molecular structure is not affected.

**Figure 6:**
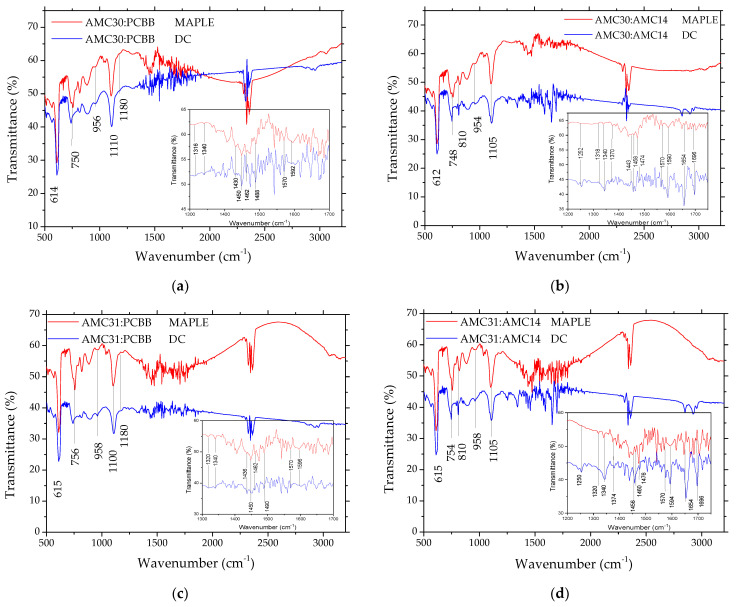
FTIR spectra of the MAPLE mixed layers by comparison with the drop cast mixed layers deposited on Si from the same solvent, 1,2 dichlorobenzene: AMC30:PCBB (**a**); AMC30:AMC14 (**b**); AMC31:PCBB (**c**); AMC31+AMC14 (**d**). The insets present the details of the FTIR spectra for AMC30/AMC31:PCBB in the spectral range 1300–1700 cm^−1^ (**a**,**c**) and AMC30/AMC31:AMC14 in the spectral range 1200–1750 cm^−1^ (**b**,**d**).

In the FTIR spectra of the mixed layer deposited on Si (Figure 6), bands were observed at ~1590 cm^−1^ and between 1435–1488 cm^−1^, ascribed to stretching of the C=C bond in the carbazole ring, between 1316–1346 cm^−1^, assigned to the C-N bond in the aromatic ring, and at 810–820 cm^−1^ and 880–890 cm^−1^, assigned to the C-H out-of-plane bending mode in the aromatic ring [19,20]. The band situated at ~958 cm^−1^ in AMC31 and at 954–956 cm^−1^ in AMC30, corresponding to the C-H out-of-plane bending vibration of trans vinyl linkage, confirmed the vinyl structure of both the polymers AMC31 and AMC30 [19,20]. FTIR spectra of the mixed layer (Figure 6) also revealed the bands assigned to the aromatic =C-H stretching vibrations, those ascribed to aliphatic CH and CH_2_ situated at a wavenumber between 2800 cm^−1^ and 3000 cm^−1^ in both AMC31 and AMC30, and the typical bands for polycarbazole at a wavenumber >3000 cm^−1^ [19,20].

The absorption bands typical for the characteristic chromophoric groups in AMC14 (Figure 6b,d) are the following [38,68,69]: 1590–1594 cm^−1^ due to C=C stretching aromatic, 1340 cm^−1^ and 1370–1374 cm^−1^ ascribed to C-N stretching, and 1250–1252 cm^−1^ assigned to –CH=CH– trans out-of-plane bending. The bands situated at 748 cm^−1^ and 810 cm^−1^ were attributed to C-H bending, and those situated at 612–615 cm^−1^ to C=C out-of-plane ring bending, and overlapped with the bands of AMC31 or AMC30 polymers situated in the same spectral range. Additionally, two very relevant bands for the chemical structure of the compound were situated at 1695 cm^−1^ due to imide C=O in-plane asymmetric stretching, and at 1654 cm^−1^ due to imide C=O out-of-plane asymmetric stretching. Between 2850 cm^−1^ and 3000 cm^−1^ were bands assigned to C–H aliphatic stretching and C–H aromatic stretching [70].

FTIR spectra of the MAPLE mixed layers containing PCBB also revealed peaks situated around 1180 cm^−1^ and 1430 cm^−1^ (Figure 6a,c), associated with the vibrations of the fullerene molecules [71,72]. This means that, during the MAPLE deposition, fullerene did not suffer any chemical degradation.

A weak impurification of the mixed layers with molecules of solvent can be taken into account, because the bands situated at 1100 cm^−1^, around 1460 cm^−1^ and 1570 cm^−1^, associated with 1,2 dichlorobenzene, were identified in the FTIR spectra [73] (Figure 6a–d). This could be explained by the volatility of the selected solvent which makes the complete removal from the film difficult.

The bands associated with the chromophoric groups characteristic of the polymers were revealed both in the layers prepared by MAPLE, implying laser technique, and by drop cast, implying the free evaporation of the solvent at room temperature. This means that the chemical structure of the polymers was not affected during laser deposition. Some characteristic vibrations were not revealed because the corresponding peaks overlapped with peaks corresponding to characteristic vibrations of the other functional group, which are situated in the same spectral region.

**Figure 7:**
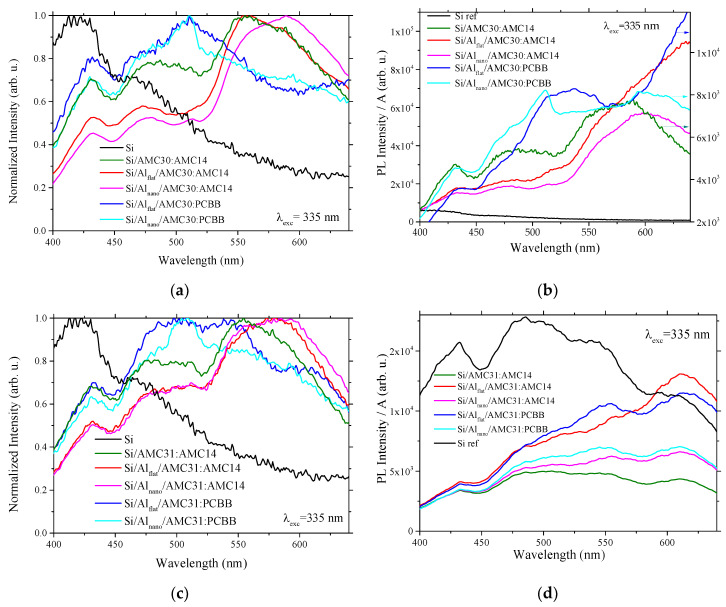
Photoluminescence spectra at excitation with λ = 335 nm of: AMC30:PCBB and AMC30:AMC14 on Al_flat_ and Al_nano_ normalized to PL maximum (**a**); AMC30:PCBB and AMC30:AMC14 on Al_flat_ and Al_nano_ divided by absorbance in reflection (**b**); AMC31:PCBB and AMC31:AMC14 on Al_flat_ and Al_nano_ normalized to PL maximum (**c**); AMC31:PCBB and AMC30:AMC14 on Al_flat_ and Al_nano_ divided by absorbance in reflection (**d**). A = Absorbance.

**Figure 8:**
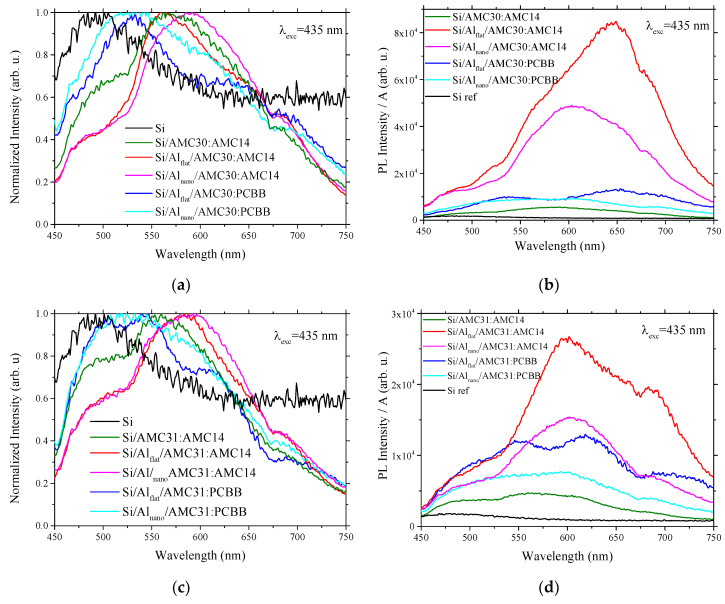
Photoluminescence spectra at excitation with λ = 435 nm of: AMC30:PCBB and AMC30:AMC14 on Al_flat_ and Al_nano_ normalized to PL maximum (**a**); AMC30:PCBB and AMC30:AMC14 on Al_flat_ and Al_nano_ divided by absorbance in reflection (**b**); AMC31:PCBB and AMC31:AMC14 on Al_flat_ and Al_nano_ normalized to PL maximum (**c**); AMC31:PCBB and AMC30:AMC14 on Al_flat_ and Al_nano_ de3vided by absorbance in reflection (**d**). A = Absorbance.

For interpretation of the emission properties of the samples, the photoluminescence signal was processed using two procedures: (a) by dividing the experimental photoluminescence values to the photoluminescence maximum value for each excitation wavelength and each sample (Figure 7a,c and Figure 8a,c) to obtain a better identification of the emission peaks’ position, and (b) by dividing the experimental photoluminescence values to the absorbance in reflection for each emission wavelength and each sample to assure a more rigorous qualitative estimation of the photoluminescence signal intensity (Figure 7b,d and Figure 8b,d).

The layer containing AMC31/AMC30 and AMC14 deposited on Si (Figure 7a–d) showed, at excitation with λ = 335 nm (3.70 eV), a specific shape characterized by the presence of peaks situated around 430 nm, 480 nm and 550 nm. The shape of the emission spectrum was not significantly changed when the mixed layer was deposited on Si covered by Al_flat_. When the mixed layer AMC30/AMC31:AMC14 was deposited on Si covered by Al_nano_, the peak situated at 550 nm was slightly red-sifted (Figure 7a,c). The peak situated at ~430 nm overlapped the wide peak attributed to Si (situated at 420 nm) and can be associated with Si substrate. The peak positioned at 550 nm could be determined by the transition from the fundamental state (S_0_) to the first singlet excited state (S_1_) in AMC14 [74,75]. In the sample realized with AMC31 and AMC14, this peak was superimposed on the peak corresponding to 0-0 electronic transition in polymer AMC31. In the sample realized with AMC30 and AMC14, the peak situated at ~430 nm overlapped the peak corresponding to 0-0 electronic transition in polymer AMC30. The emission spectrum of AMC31:PCBB showed two peaks at 500 nm and 540 nm, while the spectrum of AMC30:PCBB showed only one peak centered at ~510 nm, determined by the energetic level involved in de-excitation. The peak situated at 500–510 nm, which was present in both samples, can be associated with the emission of PCBB.

The emission spectra revealed a weakening in the intensity for the heterostructures prepared with the AMC30:AMC14 mixed layer and nanostructured Al layer, at excitation with λ = 335 nm (Figure 7b). A decrease in photoluminescence was also evidenced by the samples realized with AMC31 and AMC14 on Al nanostructures (Figure 7d). The lowest emission was obtained for the samples with a mixed layer containing PCBB, which means that fullerene determines the quenching of the luminescence. The slightly lower photoluminescence obtained by Al nanostructuring for the sample with the AMC31/AMC30:PCBB mixed layer compared to the sample with the AMC31/AMC30:AMC14 mixed layer signifies that the absorption in reflection of the sample with the mixed layer containing PCBB is higher than that of the sample with the mixed layer containing AMC14.

A similar behavior of the prepared samples was revealed at illumination with visible radiation, λ = 435 nm (2.85 eV) (Figure 8). The layers containing AMC14 deposited on Si (Figure 8a,c) showed peaks situated at 500–510 nm and 575 nm. The peak situated at~500–510 nm overlapped the peak attributed to Si (490 nm) and can be associated with the Si substrate. The peak situated at 575 nm is associated with the AMC14 non-radiative relaxation followed by radiative de-excitation from the first singlet excited state. This peak was preserved in the emission spectra of AMC30/AMC31:AMC14 deposited on Si covered by Al. A slight red shift from 575 nm to 595 nm was introduced by Al nanostructuring. Additionally, the nanostructuring reduced the emission of the mixed layers, and the weakest photoluminescence was shown by the heterostructures with a mixed layer containing the PCBB acceptor (Figure 8b,d). This behavior is a consequence of the quenching mechanism induced by fullerene derivative.

The emission spectra of the mixed layers containing AMC30 and PCBB deposited on Al_flat_ (Figure 8a) showed a peak situated at 530 nm which was correlated with the de-excitation from an energetic level of 2.34 eV, lower than the level of 2.74 eV corresponding to the first excited state in polymer AMC30. It can be correlated with emissions from the first triplet state (T_1_), which is preceded by two steps of non-radiative relaxation: (1) from the higher singlet-excited level to the first singlet-excited level (S_1_); (2) from the first singlet-excited level to the first triplet-excited state. The emission spectra of the mixed layers containing AMC31 and PCBB deposited on Al_flat_ were dominated by an emission band with two peaks situated at 510 nm and 550 nm (Figure 8c). The last peak corresponds to de-excitations from an energetic level associated with an energy of 2.34 eV, which is above the level corresponding to the first singlet-excited state in the polymer AMC31 of 2.1 eV. The de-excitation took place in two steps: (1) from a superior vibronic level to the lowest vibronic level of the first singlet-excited state by a non-radiative process; (2) from the first singlet-excited state to the ground state (S_0_) by radiative relaxation.

Thus, the sample prepared with poly(arylenevinylene)s and non-fullerene (perylenediimide) compound on Al presented stronger photoluminescence emission compared to the samples prepared from the same poly(arylenevinylene) and the fullerene derivative PCBB. Regardless of excitation wavelengths, UV or Vis, the Al electrode nanostructuring reduced the photoluminescence of the mixed layer independently of arylenevinylene polymer donor (AMC30 or AMC31) and fullerene/perylene diimide acceptor (PCBB or AMC14) (Figure 7b,d and Figure 8b,d).

**Figure 9:**
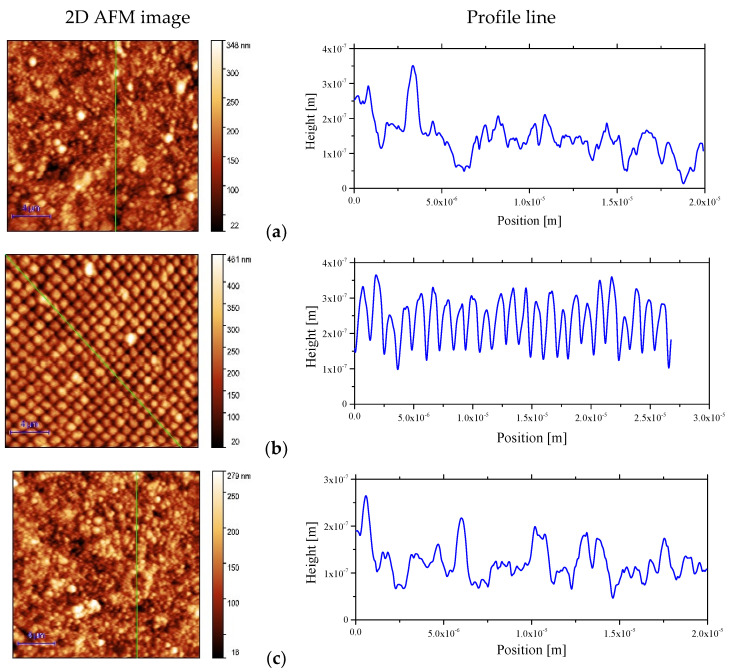

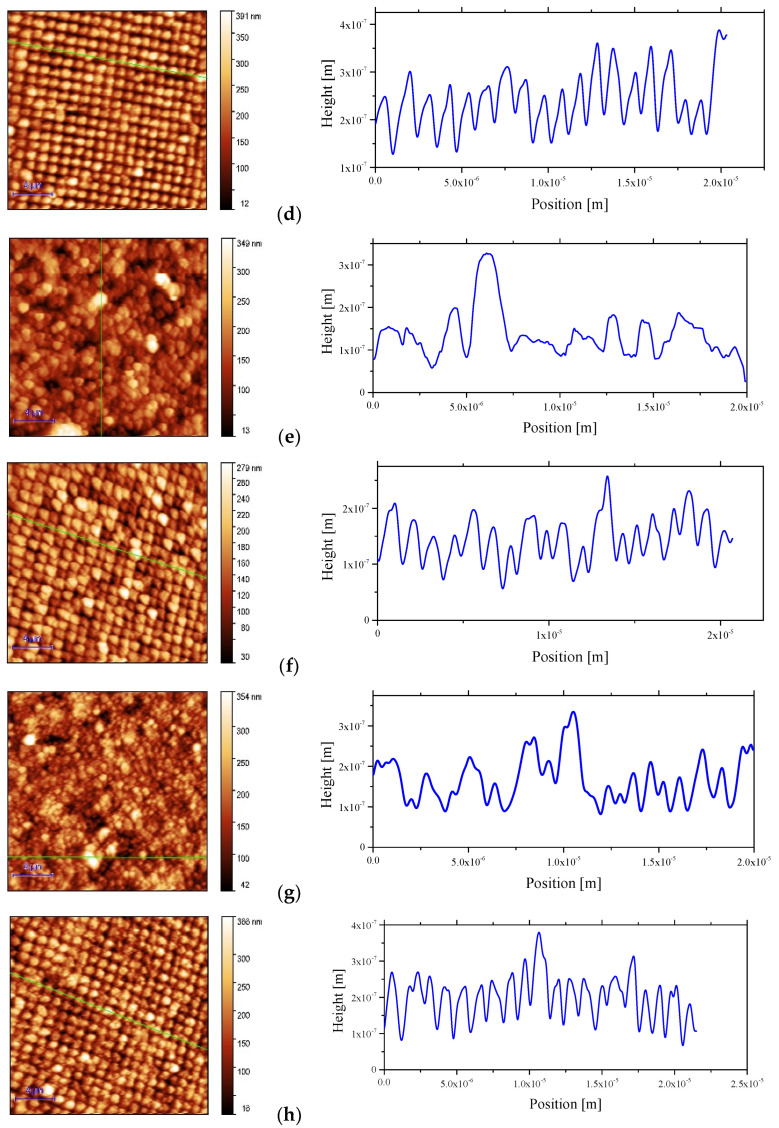
Two-dimensional AFM images and profile line of the prepared samples: Si/Al_flat_/AMC30:PCBB (**a**); Si/Al_nano_/AMC30:PCBB (**b**); Si/Al_flat_/AMC30:AMC14 (**c**); Si/Al_nano_/AMC30:AMC14 (**d**); Si/Al_flat_/AMC31:PCBB (**e**); Si/Al_nano_/AMC31:PCBB (**f**); Si/Al_flat_/AMC31:AMC14 (**g**); Si/Al_nano_/AMC31:AMC14 (**h**).

AFM analysis gave information about the surface topography of the mixed layers deposited on the nanostructured Al electrode compared to the Al flat electrode (Figure 9): the roughness of the AMC31:AMC14 mixed layer prepared with the longer conjugation length polymer increased from RMS = 44.7 nm, RA = 34.7 nm (Figure 9g) when it was deposited on Al_flat_ to RMS = 58.0 nm, RA = 46.6 nm (Figure 9h) when it was deposited on Al_nano_. The same behavior was revealed by the mixed layer prepared with the shorter conjugation length polymer, RMS = 37.5 nm, RA = 29.4 nm (Figure 9c) for AMC30:AMC14 deposited on Al_flat_ and RMS = 65.2 nm, RA = 53.6 nm (Figure 9d) for AMC30:AMC14 deposited on Al_nano_. The preservation of nano-patterning after the deposition of the mixed layer was observed on the AFM images (Figure 9b,d,f,h). The behavior of the mixed layers containing PCBB is determined by the polymer chain folding and the property of fullerene derivatives to generate clusters or aggregates of different dimensions [61,76]. The mixed layers prepared with both types of polymers and PCBB on Al_flat_ showed similar roughness: RMS = 46.4 nm, RA = 35.2 nm for AMC31:PCBB (Figure 9e) and RMS = 43.3 nm, RA = 32.9 nm for AMC30:PCBB (Figure 9a). The longer-conjugation-length polymer AMC31 suffered many folds filling the gaps between the pillars of nanostructuring, which assures a lower roughness of the layer deposited on nano-patterned Al: RMS = 42.0 nm, RA = 33.3 nm (Figure 9f). In the case of the shorter-conjugation-length polymer AMC30, the space between pillars was less filled with molecules and the roughness was higher: RMS = 67.8 nm, RA = 55.6 nm (Figure 9b).

A certain morphological regularity was observed in the samples deposited on Al_nano_. Most of the mixed layers deposited on Al_flat_ were characterized by large grains/clusters of grains of different dimension morphology (Figure 9), as revealed by the profile line, with the exception of AMC31:PCBB (Figure 9e). These mixed layers showed lower roughness, which is associated with a high reflectance. In the case of the same mixed layers deposited on Al_nano_, the multiple scattering of the radiation inside the nanostructures developed in the Al layer led to a disruptive superposition and lower reflectance associated with higher roughness (Figure 5a,b).

Another parameter which can be evaluated from the AFM data describing the height asymmetry of the surface is skewness (S_SK_). The skewness represents the asymmetry of the surface deviation from a median line or plane. In our case, this parameter could be useful in estimating how good the contact between the mixed layer and ITO electrode was. 

All the investigated films were characterized by positive values of S_SK_. (Table 1), describing the points located above the mean plane. This means that the peaks dominate on the area scanned by AFM. In this case, the collection/injection of the charge carriers was favored by the larger contact area between the ITO top electrode and the mixed layers. All the films deposited on nanostructured Al showed lower values of S_SK_ than the films deposited on flat Al, which corresponds to a smaller deviation from symmetry in the heights/valleys distribution and a smaller contact area mixed layer/ITO. The number of peaks were close to the number of valleys, meaning a symmetric surface deviation from the mean plane was obtained for the sample Si/Al_nano_/AMC30:PCBB. The highest values of S_SK_ were shown by the samples with mixed layers containing AMC31/AMC30 and PCBB deposited on Al_flat_ (Table 1), which suggests a surface topography characterized by a higher number of peaks than valleys. By replacing PCBB with the non-fullerene acceptor, AMC14, S_SK_ decreased and the number of peaks diminished, reducing the contact area with ITO.

The kurtoisis (S_KU_) parameter is a measure of the sharpness of the surface height distribution. The S_KU_ of the mixed layer deposited on Si/Al_nano_ was lower than the S_KU_ for the same layer deposited on Si/Al_flat_. This means that the mixed layer deposited on nanostructured Al was characterized by a lower number of great heights, compared to the layer deposited on flat Al. The highest values of S_KU_ were revealed by the mixed layers containing PCBB (5.1477 for AMC30:PCBB and 5.0631 for AMC31:PCBB) deposited on Al_flat_. The high values of S_KU_ correspond to spiky surfaces and were correlated with a high number of high peaks. The values of S_KU_ < 3 correspond to bumpy surfaces and means that only a few high peaks and few low valleys were identified on the scanned area. The lowest value for S_KU_ (2.6104) was obtained for the mixed layer containing the shorter conjugation polymer (AMC30) and non-fullerene acceptor AMC14, deposited on nanostructured Al.

Hence, the surface topography of the samples was characterized by the presence of morphological formations, named hillocks, of different width and height. The hillocks generated a shape of the surface showing peaks and valleys with a specific distribution, which affect the quality of the contact between the organic and ITO electrode and thus, the electrical properties of the heterostructures.

**Figure 10:**
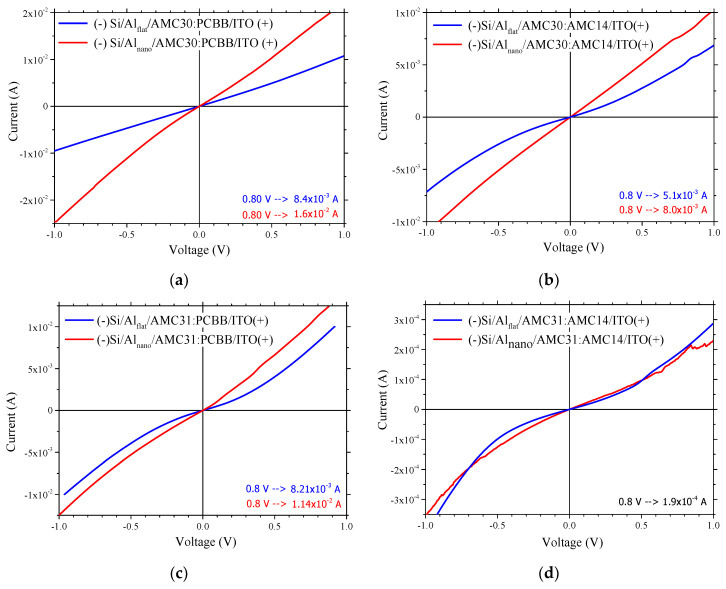
I-V characteristics in dark for the heterostructures prepared on Al_flat_ and Al_nano_ with the following bulk heterojunctions: AMC30:PCBB (**a**); AMC30:AMC14 (**b**); AMC31:PCBB (**c**); AMC31:AMC14 (**d**). Polarization is indicated in the figures.

All the prepared samples showed slightly non-linear injection contact behavior, high currents in the range 10^−4^–10^−2^ A for an applied voltage around 1 V and an increase in the current by nano-patterning of the Al electrode (Figure 10). The Al pillars penetrate the organic layer, offering a three-dimensional (3D) pathway for the charge carrier injection/collection [77]. The samples realized with fullerene derivative PCBB showed an increase in current through the nanostructuring of Al. This increase, from 8.4 × 10^−3^ A to 1.6 × 10^−2^ A at 0.8 V (Figure 10a), was slightly higher for the donor polymer AMC30, containing a carbazole unit in position (3,6) characterized by a high roughness of the mixed layer surface, compared to the increase from 8.2 × 10^−3^ A to 1.14 × 10^−2^ A at 0.8 V (Figure 10c) for the donor polymer AMC31 containing a carbazole unit in position (2,7) characterized by a lower roughness. The current was weaker (1.9 × 10^−4^ A at 0.8 V) in the heterostructures realized on Al_flat_ with AMC14 and the longer-conjugation-length polymer AMC31 (Figure 10d). In this case, the effect of nano-patterning on the current was not significant. The heterostructure with a mixed layer containing the polymers AMC30 and AMC14 on Al_flat_ showed a slight increase in the current (5.1 × 10^−3^ A at 0.8 V) (Figure 10b), compared to the heterostructure with a mixed layer containing the polymers AMC31 and AMC14 on Al_flat_ (1.9 × 10^−4^ A at 0.8 V) (Figure 10d). The nano-patterning slightly increased the current in the heterostructure with an AMC30:AMC14 mixed layer from 5.1 × 10^−3^ A to 8.03 × 10^−3^ A at 0.8 V (Figure 10b).

Based on AFM data analysis, we can anticipate that the samples Al_flat_/AMC31:PCBB/ITO and Al_flat_/AMC30:PCBB/ITO will show a better charge carrier collection/injection and higher currents. These mixed layers have a surface topography with hillocks randomly dispersed. The high S_SK_ values (Table 1), associated with an increased number of peaks, assure an increased contact area of mixed layer/ITO electrode and good collection/injection of the charge carrier. 

The I-V experimental measurements revealed that the heterostructures containing fullerene derivative, Al_nano_/AMC30:PCBB/ITO and Al_nano_/AMC31:PCBB/ITO, and the heterostructure containing a non-fullerene acceptor, Al_nano_/AMC30:AMC14/ITO, showed a significant increase in current compared to the same heterostructures on Al_flat_. The best currents were shown by the heterostructures realized with a PCBB acceptor on Al_nano_, independently of the donor polymer, AMC31 or AMC30 (Figure 10a,c). 

The electrical properties of the heterostructures deposited on Al_nano_ were not determined only by the mixed layer surface topography. This behavior can be the result of other factors, such as the position of the Al pillars in the mixed layer. Because the donor and acceptor were blended in the ratio 1:2, this increases the probability for Al pillars to be situated in the acceptor region. When Al is negatively polarized, the electrons are rejected by Al pillars and driven under the effect of the electric field towards the interfaces between the donor and acceptor, inside the mixed layer. The holes are rejected by the positively polarized ITO electrode and attracted to the donor–acceptor interfaces, favoring the electron–hole recombination, which has as a consequence a decrease in current [78]. If the Al pillars penetrate into the donor compound region, a positive effect could be obtained on the current because the probability of recombination is reduced by the lower mobility of the charge carriers [78]. This positive effect of the nano-patterning could be amplified by a better correlation between the mixed layer morphology and the periodicity of the 2D array of structures. Moreover, the very close position of the HOMO level in AMC30 and AMC14 favors the injection of the holes from AMC14 to AMC30 inside the mixed layer.

Thus, the electrical behavior of the heterostructures with a mixed layer containing PCBB is independent of polymer type (conjugation length of arylenevinylene-based polymers). For the heterostructures with a mixed layer containing AMC14, the type of polymer is important. The nano-patterning of the Al electrode has determined the higher increase in the current for the samples with mixed layers based on the fullerene derivative PCBB (Figure 10a,c). The strongest effect was revealed on the sample with a mixed layer based on the shorter-conjugation-length polymer AMC30 donor (Figure 10a). In the case of non-fullerene perylene diimide acceptor (Figure 10b,d) the best effect using nano-patterning was also obtained with the arylenevinylene-based polymer donor characterized by a shorter conjugation length and high surface roughness (Figure 10b).

The positive effect of Al electrode nano-patterning on the current value suggests that nano-patterning of Al electrodes could be used as a way to favor the injection/collection of the charge carrier. Therefore, the mixed layer deposited on the Al_nano_ electrode, containing as a donor the shorter-conjugation-length arylenevinylene-based polymer, and as an acceptor either fullerene derivative or perylene diimide derivative, Al_nano_/AMC30:PCBB/ITO and Al_nano_/AMC30:AMC14/ITO, could be used as a charge injection element in organic electronics.

## 4. Conclusions

The properties of the organic heterostructures Al/mixed layer/ITO with a mixed active layer deposited using Matrix Assisted Pulsed Laser Evaporation on flat Al and a nano-patterned Al electrode were investigated. The mixed layer was composed of arylenevinylene-based polymers with different conjugation lengths, containing carbazole units substituted at 2,7- and 3,6-positions as donors, and two different acceptors, a fullerene derivative, [6,6]-phenyl C_61_ butyric acid butyl ester, and a non-fullerene compound, N,N′-bis-(1-dodecyl)perylene-3,4,9,10 tetracarboxylic diimide, blended in the weight ratio 1:2. A 2D array of cylindrical pillars with a periodicity of 1.1 µm, diameter of 400 nm and height of 300 nm was prepared in a photoresist polymeric layer using UV Nanoimprint Lithography. This nanostructured surface was covered by the layer of Al, causing a slight change in the shape of the pillars. The edges became irregular, generating a morphology characterized by hillocks/clusters of boules with an inflorescence-like aspect. The increase in the pillars’ diameter was the result of the Al attachment both on top of the pillars and on the lateral walls of the pillars, determining an increase in the area of the pillars’ bases. The significant increase in the height of the pillars from ~300 nm without an Al layer to ~410 nm with an Al layer is the result of the difference between the thickness of the layers of molecules deposited in the upper region of the pillars and those deposited in the region at the base of the gap delimited by the pillars. Independently of the acceptor, the mixed organic layer preserved the nano-patterning of the Al substrate. 

The optical properties of the Al nano-patterns were determined, beside the particularities of nano-patterning and interference, by the resonances of the localized surface plasmons and light trapping correlated with the enhanced scattering. The reflectance of the mixed layers deposited on nano-patterned Al was reduced by the light trapping through multiple scattering inside the gaps delimited by the nanostructures.

The roughness of the mixed layer deposited on nano-patterned Al increased compared to the roughness of the same mixed layer deposited on flat Al, and was associated with high reflectance. The nano-patterning was preserved in the mixed layer, determining the light trapping by multiple scattering of the radiation on the walls and bottom of gaps situated between the pillars of the nanostructure. The reduced number of nanostructures per unit area which characterizes the 2D array led to broad and shallow reflectance oscillations compared to the same mixed layer deposited on Al_flat_.

A decrease in the emission intensity was revealed by the nanostructuring of the Al layer for the heterostructures with mixed layers including a non-fullerene acceptor at excitation both with UV and Vis radiation. Weaker emissions, associated with the quenching of the luminescence, was obtained for the samples with a mixed layer containing fullerene derivative.

All heterostructures have highlighted a slightly non-linear injector contact behavior by the I-V characteristics drawn at room temperature in dark. The electrical behavior of the heterostructures with a mixed layer containing PCBB was independent of polymer type (conjugation length of arylenevinylene-based polymers). The behavior of the heterostructures with a mixed layer containing perylene diimide acceptor was affected by the type of polymer. In this case, the best effect of nano-patterning was obtained with the arylenevinylene-based polymer characterized by a lower conjugation length (with carbazole unit substituted in position 3,6) as the donor. The effect of the contact organic/ITO is dominated by the morphology of the mixed layer and/or periodicity of the 2D array.

In conclusion, both the optical and electrical properties of the heterostructures with a mixed active layer made of poly(arylenevinylene)s and perylene diimide deposited by MAPLE are significantly affected by the nano-patterning of the Al electrode. The metallic electrode nanostructuring induced an increase in the current in the heterostructures with a mixed layer containing a donor shorter-conjugation-length polymer, independently of the type of acceptor: fullerene derivative or non-fullerene perylene diimide derivative. Thus, this type of heterostructure, Al nanostructured/short-conjugation-length arylenevinylene polymer:fullerene derivative or perylene diimide derivative/ITO could be used as a charge injection element in organic electronics.

## Figures and Tables

**Table 1:** AFM roughness parameters of the mixed layers deposited on Al_flat_ and Al_nano_: RMS, RA, S_SK_ and S_KU_.

Nr.crt	Sample	RMS (nm)	RA (nm)	S_SK_	S_KU_
1	Al_flat_/AMC30:PCBB	43.3	32.9	0.8576	5.1477
2	Al_nano_/AMC30:PCBB	67.8	55.6	0.1969	2.8009
3	Al_flat_/AMC30+AMC14	37.5	29.4	0.4623	3.7144
4	Al_nano_/AMC30+AMC14	65.2	53.6	0.2486	2.6104
5	Al_flat_/AMC31:PCBB	46.4	35.2	0.7827	5.0631
6	Al_nano_/AMC31:PCBB	42.0	33.3	0.4045	3.3806
7	Al_flat_/AMC31:AMC14	44.7	34.7	0.5882	4.2866
8	Al_nano_/AMC31:AMC14	58.0	46.6	0.3832	3.0850

## Data Availability

The data presented in this study are available on request from the corresponding author.
